# An Improved 1.5-Gigabase Draft Assembly of Massospora cicadina (Zoopagomycota), an Obligate Fungal Parasite of 13- and 17-Year Cicadas

**DOI:** 10.1128/mra.00367-22

**Published:** 2022-08-29

**Authors:** Jason E. Stajich, Brian Lovett, Cassandra L. Ettinger, Derreck A. Carter-House, Tania Kurbessoian, Matt T. Kasson

**Affiliations:** a Department of Microbiology and Plant Pathology, University of California—Riverside, Riverside, California, USA; b Institute for Integrative Genome Biology, University of California—Riverside, Riverside, California, USA; c Division of Plant and Soil Sciences, West Virginia University, Morgantown, West Virginia, USA; Vanderbilt University

## Abstract

A 1.488-Gb draft genome sequence was assembled for the fungus Massospora cicadina, an obligate parasite of periodical cicadas. The *M. cicadina* genome has experienced massive expansion via transposable elements (TEs), which account for 92% of the genome.

## ANNOUNCEMENT

*Massospora* and other Entomophthorales (Zoopagomycota) are grossly understudied due to their ephemeral and fastidious lifestyles, as well as the complicated disease and host life cycles (1–4). The recent discovery of cathinone and psilocybin in *Massospora*-infected cicadas has raised questions about their biosynthesis, which have proven difficult to answer due to unwieldy metagenomes derived from field-collected cicadas (5). The generation of high-quality genomic resources is fundamental to answering these and other questions regarding *Massospora*’s unique biology and evolution.

**FIG 1:**
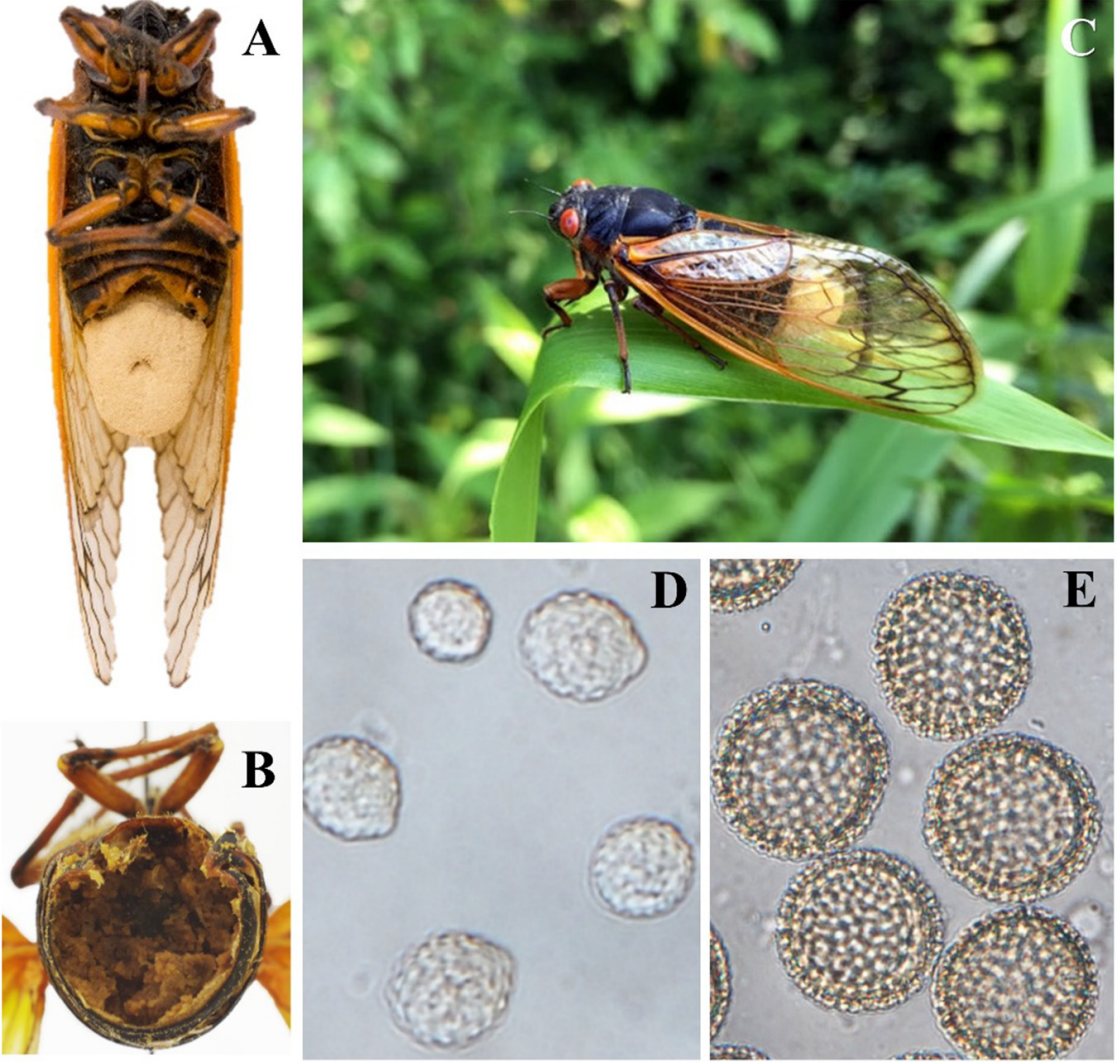
Photographs of *Massospora cicadina*-infected Pharaoh cicadas (Magicicada septendecim) and associated spore stages. (A) Adult female with conspicuous conidia “plug” protruding from the posterior end of the abdomen. (B) Adult male with inconspicuous azygospore (resting spore) infection. (C) Active male with conidial plug. (D) Close-up of *M. cicadina* verrucose (“warty”) conidia. (E) Close-up of *M. cicadina* thick-walled reticulated (“net-like”) azygospores. The photos in panels A, B, D, and E are from brood V, Morgantown, WV (2016). The photo in panel C is of a live infected cicada included in the sampling for strain MCPNR19 (ARSEF14555). Photos in panels A and B are by Cameron Stauder. Photos in panels C to E are by Matt Kasson.

Conidia and azygospores of Massospora cicadina strain MCPNR19 (ARSEF14555) were collected from *M. cicadina*-infected Magicicada septendecim in Pennsylvania in June 2019 (Fig. 1). The spores were liberated from harvested posterior fungal plugs (conidia) or by scraping abdominal walls (azygospores) of frozen infected cicada cadavers stored at −80°C. Azygospores were further isolated using 40- and 25-μm soil sieves to remove host tissue and provide sufficient fungal biomass. Genomic DNA (gDNA) was extracted from the spore pools using a fungal cell lysis and cetyltrimethylammonium bromide (CTAB) gDNA purification protocol (6). Oxford Nanopore (ONT) DNA libraries generated using the SQK-LSK109 ligation kit were sequenced on a MinION instrument with five R9.4.1 flow cells (2 for conidia, 3 for azygospores) and base called using Guppy v6.0.1-GPU (Table 1) to produce 29.7 Gb (coverage, 20×). Illumina sequencing of 1 azygospore library on a NovaSeq instrument (2× 150 bp) using a Covaris-sheared DNA library produced 26.2 Gb (coverage, ~18×). Table 1 details the library preparation, sequencing, and assembly details obtained using NanoStat v1.4.0, wtdbg2 v2.5, BBMap v38.86, and AAFTF v0.2.6 (7–11). Bacterial contamination was removed by inspection of the Blobtools2 results (12, 13), iterative taxonomic searches using MMseqs2 v13-45111 (13) with UniRef50 (14), and analysis of the fungal transposable element (TE) content (15, 16). Metagenome-assembled bacterial genomes were analyzed separately (17). A 1.488 Gbp assembly in 19,694 scaffolds was constructed from a combined read coverage of 38× (*L*_50_, 139 kb; *N*_50_, 3,261; mean GC content, 41.13%). A BUSCO v5.2.2 (18) completeness assessment identified 182 complete markers (71%) out of 255 markers in the Eukaryota Odb10 data set and 491 (65%) of 758 markers in the Fungi Odb10 data set (Table 1).

**TABLE 1:** Genome strain information, statistics, and methods for Massospora cicadina

Characteristic	Data for strain:	
	MICH 231384	MCPNR19 (ARSEF 14555)
Spore type	Conidia	Conidia, azygospores
Yr/location/cicada brood	2016/OH, USA/brood V	2019/PA, USA/brood VIII
Sampling location	I-77S rest area (Summit County)	Carnegie Museum of Natural History Powdermill Nature Reserve (Westmoreland County)
Sampling coordinates (lat, long)	41.194492, −81.624714	40.164837, −79.265278
Sequencing technology	Illumina HiSeq	Illumina NovaSeq 6000 + Oxford Nanopore MinION R9 LSK109
Assembly method	SPAdes v. 3.10.0	wtdbg2 v. 2.5, target assembly size of 1.1 Gb
Assembly polishing		wtpoa-cns using Illumina reads and Racon
Assembly contig extension		BBMap extend.sh using Illumina reads
Assembly adaptor and contamination screening		AAFTF v. 0.2.6
Genome size (Mbp)	766.56	1,488.88
GC content (%)	39.3	41.13
Scaffold *N*_50_	3,457	3,261
Scaffold *L*_50_ (bp)	67,854	139,493
No. of scaffolds	272,193	19,694
Longest scaffold (kbp)	225	1,107
No. of contigs	373,021	19,694
Avg coverage (×)	6.5	38.0
Total Illumina sequence data (Gbp)	16.6	26.22
Total Nanopore sequence data (Gbp)		29.73
Avg Nanopore coverage (×)		20
Nanopore read *N*_50_ (bp)		5,209
Longest Nanopore reads (kbp)		265, 179, 159
Nanopore read quality score^*a*^		9.9 million (85.1%) of the 11 million reads had a mean quality score of 10 (>Q10)
No. of BUSCOs (Eukaryota/Fungi Odb10 data sets [*n* {%}])		
Complete	107 (42)/301 (40)	182 (71)/491 (65)
Complete and single-copy	107 (42)/301 (40)	177 (69)/481 (63)
Complete and duplicated	0 (0)/4 (1)	5 (2)/10 (1)
Fragmented	56 (22)/110 (15)	29 (11)/74 (10)
Total no. of genes (protein-coding genes/tRNAs)	9,889 (9,510/379)	7,532 (5,435/2,079)
GenBank accession no.	QMCF00000000	JAKSWZP000000000
GenBank assembly accession no.	GCA_006912075.1	GCA_022478985.1
SRA accession no	SRR7045068 (Illumina)	SRR17553520–SRR17553524 (ONT); SRR17553525, SRR17553526 (Illumina)
BioSample accession no.	SAMN08956764	SAMN24722893
BioProject accession no.	PRJNA451007	PRJNA795459
Supporting data		
Sanger sequencing data (GenBank accession no.)	See reference 3	Representative sequences deposited as *M. cicadina* strains 8PA01 and 8PA02: 28S (MN706567, MN706568); 18S (MN706543, MN706544); EFL (MT044284, MT044285).
Specimen collection and storage		Collected alive individually in 15-mL Falcon tubes, transported on ice to lab, and stored at −80°C until sequencing. Shipped on dry ice from WVU to UCR.
Spore cleanup methods		Both: spores were aseptically removed from cicada host and placed in sterile secondary tubes for transport. Azygospores were passed through sterile 40- and 25-μm soil sieves to reduce host tissue and specifically enrich for spores.
Library prep and parameters		Illumina: NEB Ultra II FS (New England Biolabs, Ipswich, MA) (fragmentation enzyme) libraries prepared from DNA sheared with a Covaris S220 (Woburn, MA). Oxford Nanopore: constructed for R9.4.1 flow cells and the LSK109 kit. The NEBNext end-repair/dA-tailing module (E7546), NEBNext FFPE DNA repair mix (M6630), and NEBNext quick ligation module (E6056) kits (NEB) were used with the ONT SQK-LSK109 ligation sequencing kit. Sequence reads were base called using Guppy v6.0.1-GPU with the parameters “--min_qscore 7 --detect_adapter --detect_primer --trim_strategy dna.”
Contamination assessment and removal		Blobtools2 combined with manual assessment of predicted bacterial contigs using analysis of the repetitive content, which was diagnostic for fungal versus bacteria, depth of coverage, and bacterium taxonomy.
Bioinformatics methods for assembly, bacterium removal, repeat identification, and annotation		https://github.com/zygolife/Massospora_cicadina_ONT_asm (7), https://github.com/zygolife/Massospora_cicadina_MCPNR19 (8)

a Computed using NanoStat.

The genome was masked using RepeatMasker v4-1-1 (19) with Repbase (20) fungi repeats and a species-specific library generated using RepeatModeler v2.0.1 (21, 22). The repeats were screened manually to remove protein-coding genes using a DIAMOND v2.0.13 (23, 24) search of the Swiss-Prot v2021_04 database (DB) (25). The best (373 total) BUSCO-derived models were used to train the *ab initio* predictors SNAP v2013_11_29 (26) and AUGUSTUS v3.3.3 (27), with additional predictions from the self-trained programs GeneMark-ES v4.68 (28) and GlimmerHMM v3.0.4 (29). Exon evidence was generated to improve gene predictions using DIAMOND BLASTX and Exonerate v2.4.0 to align Swiss-Prot DB proteins (30). EVidenceModeler v1.1.1 (31) was used via Funannotate to generate consensus gene models with default evidence weights. tRNA genes were predicted using tRNAscan-SE v2.0.9 (32). Putative protein functions were assigned based on sequence similarity to the InterProScan v5.51-85.0 (33, 34), Pfam v35.0 (35), eggNOG v2.1.6-d35afda (36), dbCAN2 v9.0 (37), and MEROPS v12.0 (38) databases, relying on NCBI BLAST v2.9.0+ (39) and HMMER v3.3.2 (40). Secretion signals and transmembrane domains were annotated using Phobius (41) and SignalP v5.0b (42). A total of 7,532 gene models (5,453 protein-coding genes and 2,079 tRNAs) were predicted.

The genome of *M. cicadina* strain MCPNR19 is a significant improvement over the previously sequenced strain MICH 231384 (5). Similarly to the 1.018-Gbp myrtle rust genome (43) and the 1.25-Gbp soybean rust genome (44), 92% (1.369 Gbp) of the MCPNR19 genome consists of TEs, 73% of which are LTR Ty3 retrotransposons. The low predicted protein-coding gene count likely reflects gene undercalling in the absence of transcriptome sequencing (RNA-seq) data and efforts to avoid overpredicting TEs as genes (Table 1). Future work incorporating transcriptomic data is needed to validate these findings.

### Data availability.

This whole-genome shotgun project has been deposited at DDBJ/ENA/GenBank under the accession number JAKSZP000000000. The version described in this paper is version JAKSZP010000000. The sequence reads have been deposited under SRA project accession numbers SRR17553520 to SRR17553526 and BioProject accession number PRJNA795459.
